# Double venous drainage through vena cava superior in upper ministernotomy and right minithoracotomy approaches for aortic valve replacement facilitate the operation

**DOI:** 10.1186/1749-8090-8-S1-O50

**Published:** 2013-09-11

**Authors:** T Klokocovnik

**Affiliations:** 1 CV Surgery, Clinical Center Ljubljana, Slovenia

## Background

The upper mini-sternotomy and right mini-thoracotomy can confer the advantages off a smaller surgical wound for aortic valve replacement.

## Methods

236 patients were included in our study, to whom aortic valve was replaced. The purpose of this study was to examine the potential benefit of double venous cannulation through superior vena cava (SVC) for venous drainage for aortic valve replacement (AVR) in upper mini-sternotomy and right mini-thoracotomy approaches. Five to 7 cm skin incision was made at the level of third intercostal space, through which the pericardium was opened longitudinally. Shortly after the patient was fully heparinized, venous drainage was achieved by vacuum assisted (40-60 mmHg) double venous cannulation through SVC (22 French) while arterial return was established by right femoral artery or ascending aorta (standard arterial cannula. We cross-clamped the aorta and infused antegrade cold blood cardioplegia into the ascending aorta and retrograde cold blood cardioplegia into the coronary sinus to achieve cardiac arrest. The aortic valve was approached through a transverse aortotomy. The native valve was removed completely and replaced with biological or mechanical valve, secured by Teflon pledges and sutures.

## Results

Our study confirmed that even though technically challenging, upper mini-sternotomy and right mini-thoracotomy approaches for aortic valve replacement have potential advantages over conventional median sternotomy. They can offer easier exposure of ascending aorta and effective aortic valve replacement especially when using double drainage of SVC and when patients are reasonably selected for these procedures. These mini approaches were proved to be safe and efficacious.

## Conclusion

Upper mini-sternotomy and right mini-thoracotomy can be performed with standard equipment and instruments and are potentially compatible with new technologies such as suture-less aortic valve prosthesis which could be easily placed this way with a very short CPB time.

